# Extraction of Coastal Levees Using U-Net Model with Visible and Topographic Images Observed by High-Resolution Satellite Sensors

**DOI:** 10.3390/s24051444

**Published:** 2024-02-23

**Authors:** Hao Xia, Hideyuki Tonooka

**Affiliations:** Graduate School of Science and Engineering, Ibaraki University, Hitachi 3168511, Japan

**Keywords:** coastal region, storm surge, coastal levee extraction, U-Net, high-resolution satellite image, digital surface model, slope image, Matthews correlation coefficient

## Abstract

Coastal levees play a role in protecting coastal areas from storm surges and high waves, and they provide important input information for inundation damage simulations. However, coastal levee data with uniformity and sufficient accuracy for inundation simulations are not always well developed. Against this background, this study proposed a method to extract coastal levees by inputting high spatial resolution optical satellite image products (RGB images, digital surface models (DSMs), and slope images that can be generated from DSM images), which have high data availability at the locations and times required for simulation, into a deep learning model. The model is based on U-Net, and post-processing for noise removal was introduced to further improve its accuracy. We also proposed a method to calculate levee height using a local maximum filter by giving DSM values to the extracted levee pixels. The validation was conducted in the coastal area of Ibaraki Prefecture in Japan as a test area. The levee mask images for training were manually created by combining these data with satellite images and Google Street View, because the levee GIS data created by the Ibaraki Prefectural Government were incomplete in some parts. First, the deep learning models were compared and evaluated, and it was shown that U-Net was more accurate than Pix2Pix and BBS-Net in identifying levees. Next, three cases of input images were evaluated: (Case 1) RGB image only, (Case 2) RGB and DSM images, and (Case 3) RGB, DSM, and slope images. Case 3 was found to be the most accurate, with an average Matthews correlation coefficient of 0.674. The effectiveness of noise removal post-processing was also demonstrated. In addition, an example of the calculation of levee heights was presented and evaluated for validity. In conclusion, this method was shown to be effective in extracting coastal levees. The evaluation of generalizability and use in actual inundation simulations are future tasks.

## 1. Introduction

In recent years, rising sea levels and more frequent extreme weather events associated with climate change have increased the risk of coastal areas being affected by storm surges and high waves [1,2,3]. These often cause economic damage, such as damage to facilities, and threaten the safety of residents [4,5,6]. A study published in Nature highlighted the severity of these problems and provided a global analysis of the coastal areas that could be inundated by the combined effects of sea level rise, storm surges, and high waves [7]. Another study analyzed the impact of climate change on tropical cyclones and resulting storm surges, indicating that global warming may alter the magnitude and frequency of storm surges [8,9,10].

One of the effective countermeasures against these increasingly serious flood disasters, such as storm surges and tidal waves, is the installation of levees that directly prevent flooding at river mouths and coastal areas. Coastal levees are designed to withstand storm surges, tidal waves, and, in some locations, tsunamis, and play an important role in protecting the lives and property of coastal residents. The information on the location and height of levees provides important input data that greatly determine the results of simulations for predicting inundation damage, and it is essential that the information be accurate. However, coastal levee data that are uniform and sufficiently accurate for accurate inundation simulations are not always well developed [11,12,13]. Even in areas where GIS data on levees are available, the data may not correctly reflect current levees because they might have been created at an earlier date, or some of the data may contain errors or omissions. For example, in Japan, the coastal protection facility data included in the Digital National Land Information [14] is a comprehensive data set that includes coastal levee information, but the base date of the data currently available is 15 March 2012, about 12 years ago, and these data do not necessarily represent the current shape of individual coastal protection facilities and, therefore, may be erroneous [14]. In addition, there are many countries where such GIS data are not available. In this context, for example, methods have been proposed to locate levees from LIDAR data using semi-automatic algorithms [15,16], but such data are not yet fully available [17]. Therefore, improvements in the availability and quality of levee data are needed in order to improve the accuracy of inundation simulations.

Against this background, this study proposes a method of extracting levees using a U-Net model [18], a type of deep learning, using high spatial resolution optical satellite RGB images, digital surface models (DSMs), and slope images generated from DSMs, which have rapidly become popular in recent years and have high data availability.

U-Net, a type of deep learning model originally developed for biomedical image segmentation [18], has a unique structure consisting of contracted and extended paths, and it is capable of providing contextual information and accurate location data, which are essential for the analysis of complex satellite imagery and DSMs [19,20,21]. Many investigators have demonstrated the effectiveness of U-Net in satellite image analyses. For example, the task of segmenting roads from satellite images has demonstrated the ability of U-Net to extract specific features from high-resolution image data with high accuracy [22,23]. A semantic segmentation task using high-resolution satellite imagery demonstrates that U-Net is superior in processing and analyzing multimodal data for detailed geospatial analysis [24,25].

Brown et al. (2020), on the other hand, used U-Net in an extraction task for river levees, a subject similar to our study [26]. The authors selected Florida, USA, as the training area and Germany and Italy as the test areas, and input digital elevation model (DTM) data, river information, and transportation network information into U-Net to extract inland levee structures, obtaining a Jaccard coefficient of 0.73 for the training data and 0.48 for the test data. However, river levees have a large structure, and their topography is usually reflected in the DTM, while coastal levees, which are the target of extraction in this study, are basically concrete structures that do not contain information in the DTM and are difficult to detect with DTM-based methods. Therefore, this research aims to extract coastal levees by using high-resolution satellite image products (RGB, DSM, and slope), while using the same U-Net. For structurally thin concrete structures, RGB images are expected to be effective in visually identifying levees, while DSM and slope are expected to be effective in identifying levees in three dimensions.

To evaluate the superiority of U-Net in coastal levee extraction, this study evaluated it against two other deep learning models: Pix2Pix [27] and BBS-Net [28]. In order to improve the output results of U-Net, the proposed method also introduces post-processing to reduce noise from the U-Net output images. The combination of the extracted levees and DSM can also be used to calculate the levee height, and examples of this will be presented.

In the sections that follow, we first describe the coastal levee extraction method based on the U-Net model proposed in this study. Then, to validate the method, we present the results of applying the proposed method to the coastal area of Ibaraki Prefecture in Japan, discuss the potential use of the method, and conclude.

## 2. Materials and Methods

### 2.1. Proposed Method for Coastal Levee Extraction

#### 2.1.1. Method Overview

U-Net is characterized by a symmetrical U-shaped structure consisting of an encoder (downsampling) and a decoder (upsampling). The network begins by defining the number of input channels and the number of categories. The coding part of the network starts with a double convolution layer that converts the number of input channels to 64, and then gradually increases the number of channels from 64 to 128, 256, 512, and finally 1024 through four consecutive downsampling operations. These downsampling layers allow for the gradual extraction and compression of image features and serve as the basis for subsequent high-level feature interpretation. Finally, the final output is generated by the output convolution layer, which converts the number of channels to the required number of classifications. Overall, this network structure is not only efficient, but also effective in combining local and global information of the image for accurate segmentation. For levee extraction, this architecture is expected to be able to fuse satellite imagery with DSM and slope data and separate levee areas from their surroundings with high accuracy.

**Figure 1:**
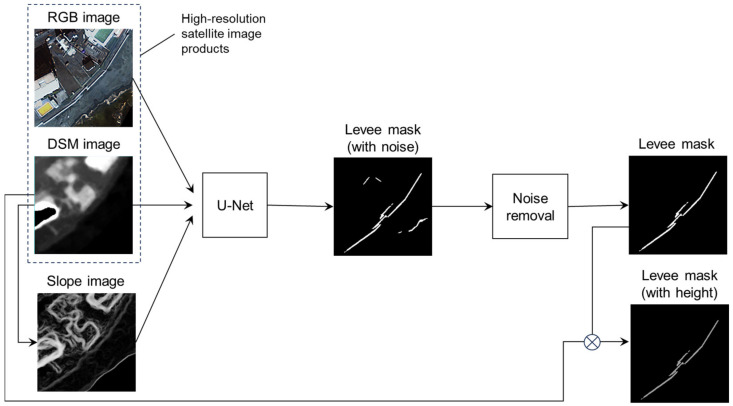
Conceptual diagram of the coastal levee extraction method.

In this study, a network structure based on the U-Net architecture was employed for coastal levee extraction. The input data are RGB color images and DSM images observed by a high-resolution optical satellite, and slope images generated from the DSM images. The output information is a levee mask image, which is a binary image in which the pixels dominated by levees have a value of 1 and the other pixels have a value of 0. The levee height can be calculated by combining the detected levee mask image with the DSM image. Figure 1 shows a conceptual diagram of the proposed levee extraction method.

#### 2.1.2. Structure and Parameters of the U-Net Model

For the loss function, the Python BCEWithLogitsLoss function with the Sigmoid activation function (Python 3.6.8; torch 1.10.1 + cu113) was used because the final layer of the network outputs logistic values. In addition, weights were employed in the loss function because the percentage of levee pixels in the output image is small. This is useful to reduce bias due to imbalance in category proportions by assigning higher weights to categories with lower proportions.

The batch size was set to 8. Dropout was used as the regularization method [29], a Dropout layer was added at the end of the two-layer convolution module, and the Dropout probability parameter was set to 0.2. Such a setting effectively prevents model overfitting and improves the generalization capability of the model. To further counteract overfitting, an early stopping strategy was employed. It monitors the dice coefficients of the validation set during the learning process and stops learning when the dice coefficients of the validation set no longer change significantly.

RAdam was employed as the optimization algorithm. It automatically adjusts the learning rate by combining the features of RMSprop and Adam, reducing the dependence of the learning rate on the hyperparameters and making learning stable and efficient [30].

#### 2.1.3. Post-Processing for Noise Removal

The following procedure was used to post-process the levee mask image output using the U-Net model for noise removal.

1. Perform segmentation based on 8 connections for pixels extracted as levees.
2. Select all segments within a predefined distance (20 pixels in this study) from the main segment with the highest number of pixels.
3. Except for the main segment and the segments selected in (2), delete the remaining segments as noise.

#### 2.1.4. Calculation of Levee Height

As a coastal protection facility against storm surge and high waves, the height of a coastal levee from sea level (mean low tide level) is more important than its height from ground level. If the DSM image used stores the geoid-corrected elevation, a levee mask image with height information can be generated by superimposing the generated levee mask image and the DSM image to obtain the DSM value of each levee pixel. Note that because the concrete structures of coastal levees are narrow, the height of the top of the levee is often not recorded in the DSM images obtained using high-resolution satellites. Therefore, in this study, the height from the DSM image was given to each levee pixel in the levee mask image, and then a local maximum filter was applied with a window size of 5 × 5.

### 2.2. Data Used

#### 2.2.1. Study Area

**Figure 2:**
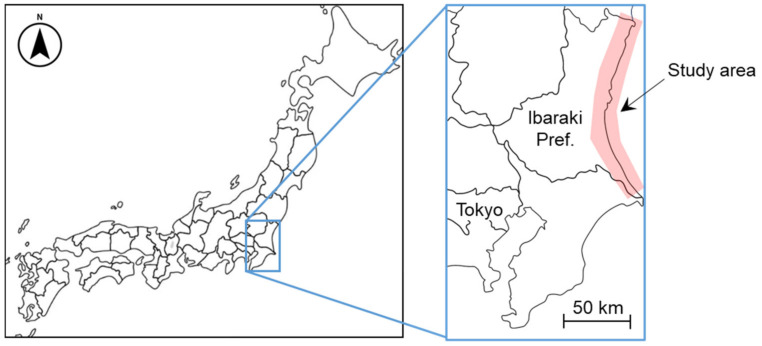
Coastal region of Ibaraki Prefecture, Japan, used as the study area.

The target area of this study is the coastal area from Kita-Ibaraki City to Hazaki City, Ibaraki Prefecture, located on the Pacific Ocean side of central Japan (see Figure 2). Ibaraki Prefecture has about 190 km of coastline and has often been hit by storm surges and tsunamis; the Great East Japan Earthquake of 11 March 2011 inundated about 25.4 km^2^ of the region with tsunami, damaging nearly 5000 houses and claiming six lives, in addition to significant economic losses [31].

#### 2.2.2. Input Images to U-Net

The high-resolution RGB images used in this study were AW3D orthorectified-sharpened image products [32], created by combining Maxar’s high-resolution satellite images taken between 19 December 2018 and 1 January 2023, with a spatial resolution of 50 cm. Table 1 lists the high-resolution satellite images used. Three-band RGB color images, excluding the IR band, were used in this study. As shown in Table 1, the high-resolution satellite images include a variety of seasons, so it is expected that there is some generalization to sunlight incidence conditions.

The high-resolution DSM images used were AW3D Enhanced products provided as the highest precision DSM/DEM, generated from Maxar’s high-resolution images [33]. The resolution of the product used in this study is 1 m in most areas and 0.5 m in some areas. These DSM values are elevation values corrected by the Earth Gravitational Model 2008 (EGM2008) [34]. Here, to emphasize the DSM values near the levees, the range of 0 to 30 m covering the DSM values in the area around the coastal levees in the study area was scaled from 0 to 255, and all pixels with DSM values greater than 30 m were given a value of 0. The resolution of the DSM images was then resampled to match the resolution of the high-resolution RGB images at 50 cm.

The slope image values were first calculated for each pixel from the DSM image obtained above using the following formula:

(1) $$slope=\arctan\left(\sqrt{\left({\left[dz/dx\right]}^{2}+{\left[dz/dy\right]}^{2}\right)}\right),$$

where *dz*/*dx* and *dz*/*dy* are the rate of change of the DSM values from the pixel of interest to its neighbors in the horizontal and vertical directions, respectively. The obtained slope values were scaled so that the minimum and maximum values were 0 and 255, respectively, and the integer-transformed values were used as the pixel values of the slope image.

#### 2.2.3. Levee Mask Images for Training

**Figure 3:**
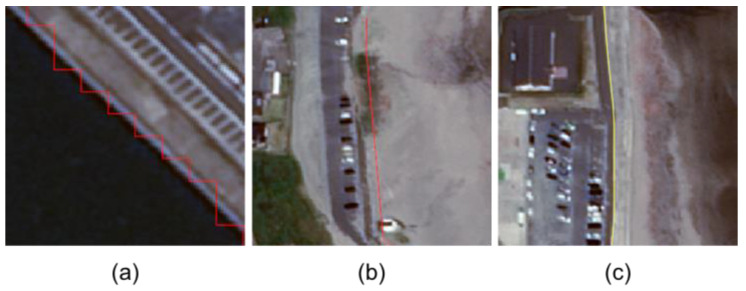
Examples of inaccuracies seen in the prefectural levee data: (**a**) is an example of an incorrectly shaped levee—a straight levee has a zigzag shape as shown in red; (**b**) is an example that includes a non-existing levee—satellite imagery and Google Street View confirm that there is in fact no red levee at this location; and (**c**) is an example of a levee that does not exist—the yellow levee is not included in the prefectural levee data.

The output image of the U-Net model is a levee mask image. The levee mask images for learning were manually created by referring to the Coastal Levee Structures Data produced by the Department of Agriculture, Forestry, and Fisheries and the Department of Public Works, Ibaraki Prefectural Government (hereafter referred to as “prefectural levee data”), Google Earth images, and Google Street View. The prefectural levee data were provided in Shapefile format and are not publicly available. The reason for not using the prefectural levee data as-is is that it contains some inaccuracies. Figure 3 shows three examples of inaccuracies found in the prefectural levee data.

#### 2.2.4. Subimage Sets

**Figure 4:**
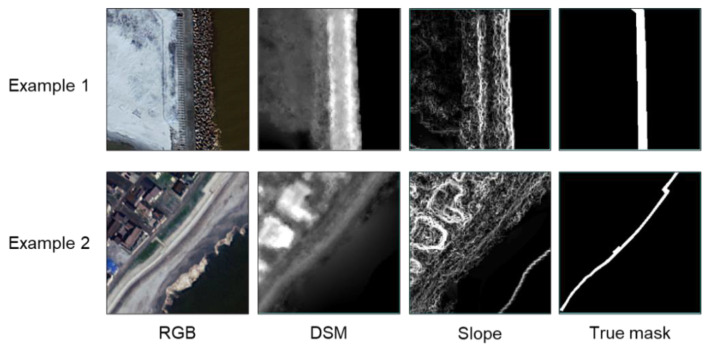
Examples of subimage sets.

The size of the subimages used for learning was 256 pixels × 256 pixels (128 m × 128 m ground size). Then, a total of 376 subimage sets containing levees were cut and prepared from the study area, with each subimage set consisting of an RGB image, a DSM image, a slope image for input, and a levee mask image for output. Figure 4 shows examples of the subimage sets. Next, data augmentation, including rotation, flipping, and sharpening, was performed on these sets to increase the number of subimage sets to 3008. Then, from these, 2708 sets were used for learning and 300 sets for validation. In addition to these, 30 subimage sets were also prepared for testing, cut from the study area. Here, the data for testing included not only the area including the levee, but also the area not including the levee.

### 2.3. Learning and Evaluation

#### 2.3.1. Evaluation Cases

In order to investigate the effect of inputting DSM and slope images as well as RGB images, we evaluated the proposed method in the following three cases:

- Case 1: Only RGB images were input;
- Case 2: RGB and DSM images were input;
- Case 3: RGB, DSM, and slope images were input.

In addition, the effect of post-processing on noise removal was evaluated by comparing the results with and without post-processing.

#### 2.3.2. Deep Learning Models to Be Compared

In this study, Pix2Pix and BBS-Net were used as models for comparison with U-Net. Comparisons of these models were made without noise removal post-processing.

Pix2Pix is one of the image-to-image transformation models and is a type of conditional adversarial generative network (CGAN). It is known that this network can be used for a variety of tasks [27]. In this study, the batch size was set to 8, and the original parameters of the Pix2Pix network were used for the learning rate and other parameters. However, since the original code does not support more than four input channels, the code was modified to allow for DSM and slope input in addition to RGB images.

BBS-Net is a network for RGB and depth (RGB-D) object detection that features a unique bifurcated backbone strategy [28]. That is, it splits multi-level features into two distinct groups: high-level features that focus on information and low-level features that focus on detail refinement. The incorporation of a depth extension module significantly improves the use of depth information in the network and enables more accurate object detection. In this study, a new slope channel was added to BBS-Net’s RGB and DSM channels to allow for RGB, DSM, and slope inputs. The learning rate was set to 1 × 10^−4^, and the original values were used for the other parameters.

#### 2.3.3. Evaluation Indices

The levee extraction accuracy was evaluated using the Matthews correlation coefficient (MCC) [35], structural similarity (SSIM), and intersection over union (IoU).

The MCC is an index that considers all four cases, true positive (*TP*), false positive (*FP*), true negative (*TN*), and false negative (*FN*), and is represented by Equation (2).

(2) $$MCC=\frac{TP\times TN-FP\times FN}{\sqrt{\left(TP+FP)(TP+FN)(TN+FP)(TN+FN\right)}},$$

where an MCC of +1 indicates a perfect prediction, an MCC of −1 indicates a perfect inverse prediction, and an MCC of 0 indicates a random prediction.

SSIM is a measure of similarity that focuses on changes in structural information, brightness, and contrast between two images, and is particularly useful for assessing the quality of segmentation that preserves the underlying structure of the original image; SSIM is shown in Equation (3).

(3) $$SSIM(x,y)=\frac{\left(2\mu_{x}\mu_{y}+C_{1})(2\sigma_{xy}+C_{2}\right)}{\left(\mu_{x}^{2}+\mu_{y}^{2}+C_{1})(\sigma_{x}^{2}+\sigma_{y}^{2}+C_{2}\right)},$$

where *x* and *y* are vectors whose elements are each pixel in a window in the image before and after encoding, respectively; *μ* is the mean pixel value in each window; *σ_x_* and *σ_y_* are the standard deviations of pixel values in the same window; and *σ_xy_* is the covariance between *x* and *y*. *C*_1_ and *C*_2_ are constants for stabilization.

IoU (or the Jaccard coefficient) is an index that directly evaluates the accuracy of segmentation by quantifying the overlap between the predicted segment and the actual segment; IoU is expressed by Equation (4).

(4) $$IoU=\frac{Intersection}{Union}$$

where *Intersection* is the number of pixels in common between the predicted and actual segments, which, in this study, is the number of pixels that both segments contained in the same levee structure. *Union* is the total number of pixels of the predicted and actual segments minus *Intersection*.

#### 2.3.4. Evaluation of Levee Height

The shapefile of the prefectural levee data contains the levee height as an attribute. The heights of the levee mask images obtained by the proposed method were compared with these attribute values to evaluate their validity.

## 3. Results

**Figure 5:**
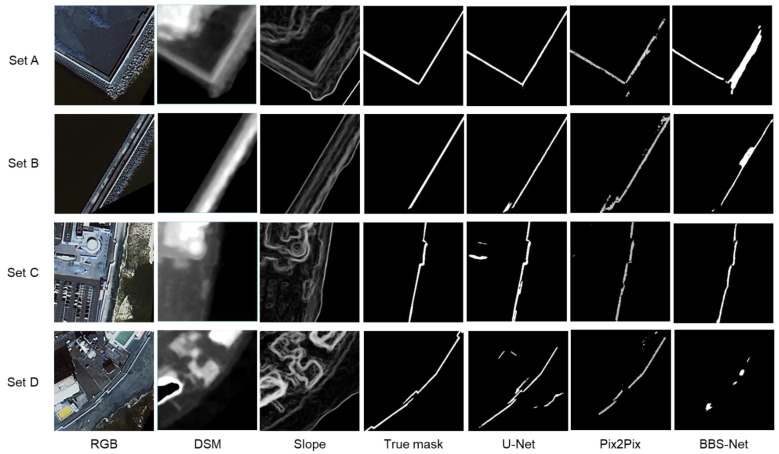
Levee mask images extracted by U-Net, Pix2Pix, and BBS-Net (without noise removal) in Case 3 for subimage sets A–D. The input images and the true levee mask image are also shown.

Figure 5 compares each input image, the true levee mask image, and each output image in Case 3 (RGB + DSM + slope) of the three models (U-Net, Pix2Pix, and BBS-Net) for the four test image sets A–D. No noise removal post-processing was applied to any of them. These images contain linear features such as shorelines and roads that resemble levees as well as actual levees, but, overall, it can be seen that the levees are selectively extracted in all models. For Set A, U-Net shows results similar to the true mask image, but Pix2Pix shows some missing and BBS-Net shows some over-detected pixels; Set B shows a similar trend. For Set C, BBS-Net shows better results and U-Net shows some over-detected pixels on the left side. In Set D, U-Net shows some undetected and over-detected pixels, while Pix2Pix shows no over-detection, but more undetected pixels than U-Net. BBS-Net also fails to detect levees.

Table 2 shows the average values of MCC, SSIM, and IoU for the three models in Case 3 using 30 sets of test images. For all indicators, the U-Net results are the best, which is consistent with the trend of visual evaluation in Figure 5. Based on the above results, in the following sections, we will show the results when the deep learning model is U-Net.

**Figure 6:**
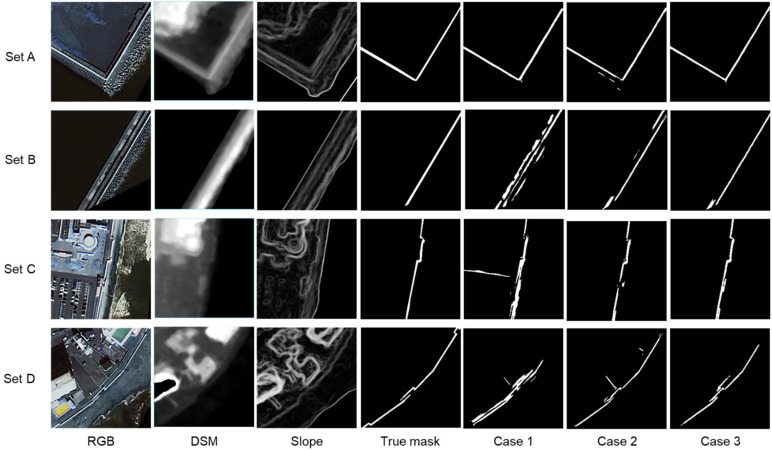
Extracted levee mask images for Cases 1–3 for subimage sets A–D (model: U-Net; noise removal: yes). The input images and the true levee mask image are also shown.

Figure 6 shows the input images, the true levee mask image, and the levee mask images derived in Cases 1 to 3 for the four test image sets A to D. The model used was U-Net, and noise removal post-processing was applied. A is an example where the levee was extracted well in all cases. B is an example where the addition of the DSM image improved the accuracy, and while Case 1 contains many non-levee pixels, Cases 2 and 3 are relatively good at extracting the levee. C and D also show examples of improved accuracy due to the addition of the slope image, and it can be seen that the non-levee pixels seen in Case 2 were removed in Case 3.

Table 3 shows the average values of MCC, SSIM, and IoU for the 30 sets of test images. For all indices, Case 3, in which RGB, DSM, and slope images were input, shows the highest performance, while Case 1, in which only the RGB image was input, shows the lowest performance. In the case of MCC, Case 3 has the highest value of 0.674.

**Figure 7:**
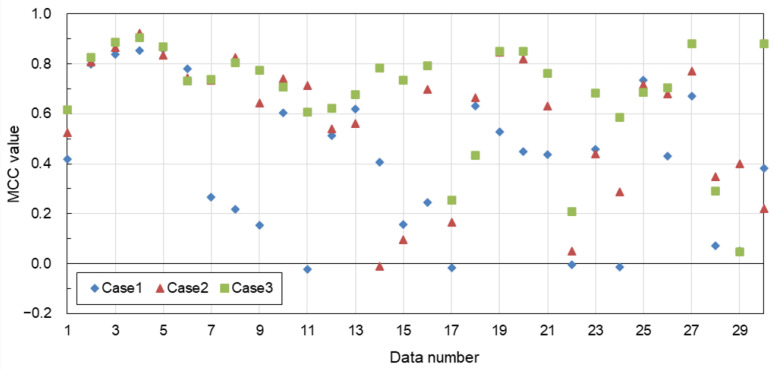
MCC values for each test image in Cases 1–3 (model: U-Net; noise removal: yes).

Figure 7 is a plot showing the MCC in Cases 1–3 for each of the test images from No. 1 to 30. It can be seen that Case 3 has the highest MCC overall. However, there are five test images (Nos. 17, 18, 22, 28, and 29) with MCCs below 0.5, even in Case 3.

**Figure 8:**
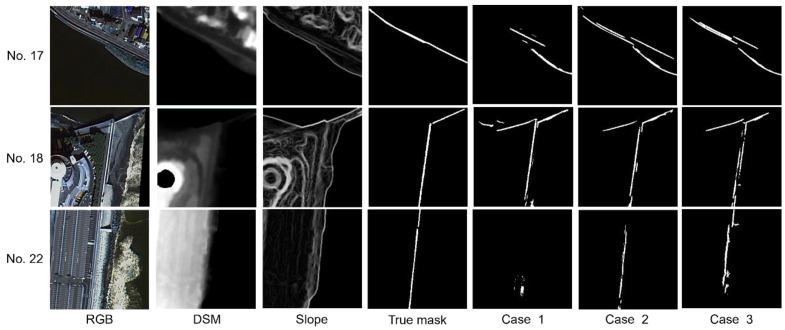
Results for No. 17, No. 18, and No. 22 with low MCC in Case 3 (model: U-Net; noise removal: yes).

Figure 8 shows the output results for Nos. 17, 18, and 22 in Case 3 of the test image sets with low accuracy shown in Figure 7. For No. 17, the correct levee mask image shows that the levee is located close to the road, away from the sea, but the presence of structures other than the levee near the sea and the large slope value near the boundary between the sea and the land are likely error factors. For No. 18, the MCCs of Case 1 and Case 2 are 0.633 and 0.667, respectively, while the MCC of Case 3 is 0.435. This may be due to the fact that the levee mask image in Case 3 had a discontinuity in the levee due to the slope image, which caused some levee pixels to be removed during post-processing. For Case 22, the MCC is −0.01 for Case 1, 0.10 for Case 2, and 0.21 for Case 3, which was well below the average MCC in all cases. According to Google Street View, the levee structure at this location is much smaller than at other locations, which could be a possible error factor.

**Figure 9:**
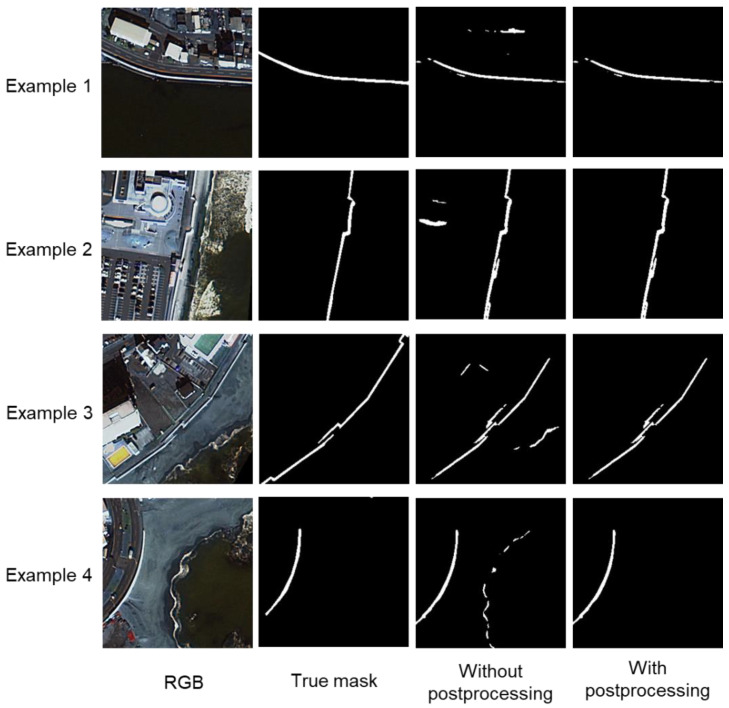
Comparison of levee mask images without and with post-processing in Case 3 (model: U-Net; noise removal: yes).

Figure 9 shows a comparison of the cases without and with noise removal during post-processing, both with all input data (Case 3). These show how post-processing removes pixels that are clearly not levees. Table 4 shows the evaluation index values for all test data without and with post-processing. The post-processing case shows higher values for all indices, indicating that the post-processing is effective.

**Figure 10:**
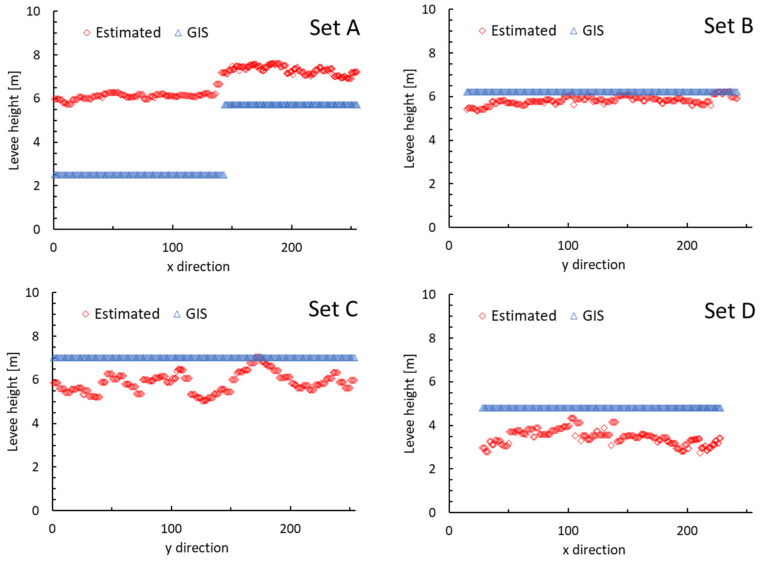
Comparison of heights estimated using the proposed method for the subimage sets A–D with those recorded in the prefectural levee GIS data.

Figure 10 compares the levee heights in the prefectural levee data with those estimated by giving the heights from the DSM image to the levee mask image in Case 3 and applying the local maximum filter for subimage Sets A to D in Figure 6, where Sets A and D show profiles moving in the x-axis (left to right) through the levee segment, and Sets B and C show profiles moving in the y-axis (top to bottom) through the levee segment. In Set B, the estimated and GIS values are nearly consistent. In Sets C and D, the estimated values are slightly smaller than the GIS values, and this is reasonable because the resolution of the DSM images used is basically 1 m and the DSM images do not necessarily include the tops of the levees. On the other hand, Set A, which is the reclaimed area of the port, conversely shows values that are 1 to 4 m larger than the GIS values, but since the GSI map [36] shows the elevation of the ground around the levee as 2 to 3 m, the left side of the levee segment as about 4.4 m, and the right side as about 6.5 m, the GIS data values are slightly smaller, and the DSM values are likely to be slightly larger.

## 4. Discussion

In this study, we proposed a method of inputting high spatial resolution optical satellite RGB images, and the digital surface models (DSMs) and slope images generated from them, into a U-Net model to output the primary results of levee mask images, which are then post-processed to remove noise to obtain the final levee mask images. The applicability and accuracy of the model were evaluated for the coastal area of Ibaraki Prefecture, Japan.

In the first comparison between the deep learning models, the U-Net approach employed in the proposed method showed better extraction performance than Pix2Pix and BBS-Net. In addition, the test images contained many coastlines and roads with linear features similar to levees, but the proposed method selectively extracted levees. This may be due to the effect of adding 3D information from DSM and slope in addition to the visual information from the RGB images. In fact, a comparison using 30 sets of test data for three patterns of input data—RGB image only (Case 1), RGB image and DSM image (Case 2), and RGB image, DSM image, and slope image (Case 3)—showed that the average MCC was 0.417 for Case 1, 0.576 for Case 2, and 0.674 for Case 3, indicating that it is effective to input DSM and slope images in addition to RGB images. On the other hand, there were some results with a relatively low MCC, even in Case 3, and these were cases where the levee structure was small or there was another structure nearby. Regarding the post-processing noise removal, the average MCC without post-processing was 0.648 and with post-processing was 0.674 for Case 3, indicating the effectiveness of post-processing.

A method was also proposed to estimate the height of the extracted levee by giving the levee pixels in the extracted levee mask image the DSM value from the DSM image and then applying a local maximum filter. In an evaluation using four test images, one image showed a close match between the estimated height and the height of the prefectural levee data. On the other hand, in two images, the estimated height was generally smaller than the height of the prefectural levee data. This may be because the resolution of the DSM images used in this study was basically 1 m, and the tops of some levees were not included as DSM pixels. This suggests that the resolution of the DSM images should be as high as possible for the proposed method. In the one remaining image, the estimated height was about 1 to 4 m greater than the height of the prefectural levee data, but an evaluation with other map information suggested that this may be due to the slightly smaller height of the prefectural levee data and slightly larger DSM values. The performance of the height assignment to the extracted levee mask depends on the relationship between the levee width and DSM resolution and tends to underestimate, but the results are generally expected.

Making a comparison with Brown et al.’s study [26] is difficult due to differences in the extraction target (their target was river levees, while our target was coastal levees), the type of input data, and the test data, but an evaluation with the test data showed that their Jaccard coefficient was 0.48, while our value (IoU) was 0.542, which is slightly higher. This suggests that the proposed method performs as expected, considering that the target of extraction in this study was small concrete structures not included in the DTM. However, accurate levee information is required for inundation simulations: if there is a levee, but the levee data are missing, the simulations will overestimate inundated areas. On the other hand, if there is no levee, but it is assumed that there is a levee, the inundation area will be missed. From this point of view, the accuracy required for levee extraction should be 100%, but this is difficult to achieve via satellite or DSM images using any method. Therefore, it is recommended that the levee mask data from the proposed method be used to discover differences with the input levee data and to create more optimal levee data, rather than being used as-is in the inundation simulation. For example, the prefectural levee data referred to in creating the correct levee mask image this time was found to be partially erroneous as a result of evaluation using satellite imagery and Google Street View. Possible causes for this include cases where levee construction was performed after the data were created, or errors in the work of the data creator. Similarly, the coastal protection facility data [14] currently provided as part of the Digital National Land Information are from March 2012 and differ from the current status in some locations, and also contain errors. On the other hand, the satellite images used in this study for levee extraction were observed between 2018 and 2023 and are believed to more accurately reflect the current levee situation. Therefore, by using the proposed levee extraction model and high-resolution satellite image products with appropriate timing and resolution, it should be possible to detect errors in the levee GIS data used for inundation simulations. If accurate levee data are already available, it may be possible to detect levee failure due to storm surge or earthquake by applying the proposed method.

Since the model was trained using images from various seasons in this study, it appears to have a certain degree of generalizability to sunlight illumination conditions. On the other hand, since the learning was conducted for the coastal area of Ibaraki Prefecture, it should be applicable if the type of levee structure is similar to that of the study area, but if it is completely different, it may be necessary to train the model for the target area used. A more detailed evaluation of generalizability is a future research issue.

## 5. Conclusions

The U-Net model of coastal levee extraction proposed in this study performed best when RGB, DSM, and slope images from high-resolution satellites were used as input. Noise removal via post-processing was also effective. The method of estimating levee height from the combination of extracted levees and DSMs generally performed as expected, although there was a tendency to underestimate depending on the relationship between the levee width and DSM resolution. However, there were instances where the accuracy decreased when the levee structures were small or when other structures were nearby. Therefore, it is recommended that the levee mask data from the proposed method be used to discover differences with the input levee data and to create more optimal levee data, rather than being used as-is in the inundation simulation. The next step is to evaluate the applicability of the method to other regions and to use the method in actual inundation simulations.

## Figures and Tables

**Table 1:** List of high-resolution satellite images used.

Subarea Name	Satellite	Ground Resolution	Observation Date
Hazaki	GeoEye-1	0.5 m	1 January 2023
Kashima	WorldView-2	0.5 m	10 May 2021
Nakaminato and Oarai	WorldView-3	0.5 m	19 December 2018
Tokai	WorldView-2	0.5 m	2 July 2022
Kita-Ibaraki and Hitachi	WorldView-2/3	0.5 m	21 April 2021, 5 August 2021, 25 November 2021

**Table 2:** Values of each evaluation index obtained using U-Net, Pix2Pix, and BBS-Net (without noise removal) in Case 3.

Index	U-Net	Pix2Pix	BBS-Net
MCC	0.648	0.521	0.533
SSIM	0.950	0.939	0.946
IoU	0.507	0.368	0.390

**Table 3:** Values of each evaluation index obtained in Cases 1–3 (model: U-Net; noise removal: yes).

Index	Case 1 (Only RGB)	Case 2 (RGB + DSM)	Case 3 (RGB + DSM + Slope)
MCC	0.417	0.576	0.674
SSIM	0.926	0.951	0.958
IoU	0.309	0.447	0.542

**Table 4:** Evaluation index values without and with post-processing in Case 3 (model: U-Net; noise removal: yes).

Index	Without Post-Processing	With Post-Processing
MCC	0.648	0.674
SSIM	0.950	0.958
IoU	0.507	0.542

## Data Availability

Data are contained within the article.
